# Bibliographical Notices

**Published:** 1838-04-25

**Authors:** 


### BIBLIOGRAPHICAL NOTICES.

*An Experimental Investigation into the Functions of the Eighth Pair of Nerves, or the Glosso-Pharyngeal, Pneumogastric, and Spinal Accessory, by* JOHN REID, M. D. Fellow of the Royal College of Physicians of Edinburgh, Lecturer on the Institutes of Medicine, formerly Demonstrator of Anatomy, &c. [From the Edinburgh Medical and Surgical Journal, January, 1838.]

(Concluded from page 131.)

PNEUMOGASTRIC NERVE. That part of the trunk of this nerve which lies in the neck, was exposed, in thirty animals, and when pinched, cut, or even stretched, *indications of severe suffering*, were given in almost every instance. The sensibility of this nerve has been noticed incidentally by Haller, Brunn, Dupuy, and Dumas.

*Pharyngeal Branches.*—In detailing the experiments upon the glosso-pharyngeal, sufficient evidence was adduced to prove the pharyngeal branch of the par-vagum, to be a *motor nerve*, distinct convulsive movements of the *constrictors* of the pharynx, stylo-pharyngeus, and the muscles of the soft palate, being caused by irritating it; and our author thinks that we are justified in inferring

"that this is the principal, if not the sole motor nerve of these parts."

"I find, that I have notes of the observations made upon the effects of pricking, cutting, and tying these nerves in seven dogs. In four of these it is expressly stated that there was not the slightest indication of suffering; in two, that there were no decided indications of suffering; and that in one, there was undoubted indications of suffering. * * * * It is quite possible that if this animal, instead of being kept alive for further observation, had been killed at the time, and the nerves carefully dissected, some unusual arrangements of the nervous twigs might have accounted for this difference; for in the other six the nerves were, as I have stated, pricked, cut, and tied, and yet no decided evidence of pain showed itself." p. 134.

To determine the effects of the section of the pharyngeal branch upon the function of deglutition, it was cut across on both sides, and a portion removed, in five dogs. In three, satisfactory observations were made. In all, the integrity of this function was sensibly affected, and this was manifested exactly in the same manner—violent, numerous, and prolonged movements in the pharynx. Whilst, then, the sensations of the pharynx and fauces depend upon the glosso-pharyngeal nerve, the pharyngeal branch of the *par-vagum* is the motor nerve of the same parts. "This view of the functions of the nerve, is supported by their ultimate distribution upon the pharynx and fauces."

*Superior Laryngeal Nerve.*—From numerous experiments tried upon animals whilst living, and also immediately after death, our author concludes that:

"One only, of the intrinsic muscles of the larynx, receives its motor filaments from the superior laryngeal, viz: *the circo-thyroid* muscle; and that consequently, the only change which this nerve can produce on the *larynx*, as a motor nerve, is that of approximating the cricord to the thyroid cartilage—in other words, of shortening the larynx." p. 140.

"That the superior laryngeal furnishes all, or at least nearly all, the *sensitive* filaments of the larynx, and also some of those distributed upon the *mucous surface* of the pharynx." p. 145.

"That the inferior laryngeal, or recurrent, furnishes the *sensitive* filaments to the upper part of the trachea; a few to the *mucous surface* of the pharynx, and still fewer to the *mucous surface* of the larynx.

"That when any irritation is applied to the mucous membrane of the larynx in a healthy state, this does not excite the contraction of the muscles which move the arytenoid cartilages, by acting *directly* upon these through the mucous membrane, but that this contraction takes place by a *reflex action*, in the performance of which the superior laryngeal is the *sensitive*, and the inferior laryngeal is the *motor* nerve." p. 146.

To the question, "*Does Section of the Laryngeal Nerves, prevent the ingress of the food into the Larynx during Deglutition?*" Dr. Reid answers that the animals swallowed both solids and liquids without inconvenience, and on careful examination after death

"Not the slightest trace of food could be detected in the air passages. From these experiments it would appear, that if the closing muscles of the glottis, can, as Magendie has shown, prevent the entrance of food into the larynx after removal of the epiglottis, the epiglottis can, on the other hand, prevent the ingress of food into the larynx, when the movements of all the muscles which diminish the size of the glottis, have been suspended by section of the laryngeal nerves." p. 146.

*Œsophageal Branches of Par-Vagum.*—Mechanical and chemical irritation of the trunk of the *par-vagum*, in an animal recently killed, induced violent muscular contractions along the whole length of the œsophagus. The tube became shortened, and its calibre diminished. These movements reached, also, to the *cardiac* extremity of the stomach, from which they extended, somewhat slowly, over a greater or less extent of that viscus.

A portion of the pneunogastric was removed on both sides, in two rabbits, above the origin of the superior laryngeals. The animals had been made to fast from sixteen to twenty hours; parsley being placed before them, they commenced eating it with avidity, and the first mouthfuls were readily swallowed. As they continued to eat, their breathing became distressing, and they seemed very uneasy. Efforts to vomit were made. After a while the *dyspnœa* abated, and they returned to their food, but the same phenomena speedily returned, forcing them to desist. Both the rabbits died. On dissection the œsophagus was found enormously distended, and compressing the trachea. The stomach was not unusually full. The larynx, trachea, and bronchi contained large quantities of the masticated parsley. "In some parts of the lungs, it was found in the minutest air cells, and one or two portions were perfectly dense, from the quantity which they contained."

"We believe that in these two experiments the first mouthful was carried into the upper portion of the *œsophagus* in the usual manner, but as the muscular movements of this tube had been suspended, it remained there. As additional mouthfuls arrived they propelled forward those which preceded them, so that after a while these formed a column of food reaching from the lower part of the pharynx to the stomach. As the difficulty of propelling it into the stomach increased, the œsophagus became more distended, and pressed upon the trachea, and thus prolonged the *dyspnœa* and uneasiness. The difficulty of propelling the food still increasing, it accumulated in the *pharynx* and passed into the open *larynx.*" p. 152.

The late Mr. Broughton and Dr. Wilson Philip having contended that the presence of the parsley in the œsophagus, depended upon ineffectual efforts of the animal to throw it off the stomach, and as, in the two experiments just mentioned, attempts at vomiting were made, Dr. Reid resolved to ascertain satisfactorily the truth of this assertion; for this purpose the previous experiments were repeated, the animal being killed "before it had given the slightest indications of any effort to vomit." A precise condition of the parts resulted in these cases.

"We believe that these experiments prove quite satisfactorily, that the *œsophagus* is distended before the *stomach*, and also fully bear out the explanation of the phenomena, arising from the retention of the food in this tube, which we have given above." p. 154.

*Pulmonary Branches of the Par-Vagum.*—In fourteen animals, Dr. Reid removed a portion of the *par-vagum* on one side, in order to determine the effects of section of that nerve upon the lungs, and in no instance did he observe any morbid structural change which he could attribute to lesion of the nerve. In all the experiments a sufficiently large portion of the nerve was removed to prevent re-union. The animals were permitted to live from four days to six months. This invalidates the statements of Swan and Magendie.

Physiologists of the present day generally concede the persistence of the respiratory movements after the division of both pneumogastrics, but explain it on very different principles. To those who contend for the exclusiveness of volition in this function, the fact would appear to lend some countenance; but as this view is, from other reasons, indubitably untenable, we shall not stop to discuss the point. The other class, who believe that respiration depends upon impressions made originally on the mucous membrane of the lungs, and reflected upon the *medulla oblongata*, must acknowledge some modification in the usual process, when they see it maintained after section of the *par-vagum.* Brachet has asserted, with more ingenuity than truth, that breathing continues after the nerves have been cut, from the result of habit! Marshall Hall believes, that when this condition occurs, the function is no longer *excito-motory*, but is now transformed into a cerebral, or voluntary act. From these hypotheses, let us turn to Dr. Reid's conclusions:

"I am satisfied that these respiratory movements will not only go on regularly and vigorously for hours together in kittens one or two days old, but that they will also proceed in animals deprived of all volition by a small dose of prussic acid. Thinking that it might be possible, though certainly not probable, that the impressions made at the lungs might in these cases reach the *medulla oblongata*, through the free-anastomoses between the laryngeal nerves at the larynx, (for in the first experiments I cut the nerves, as is generally done, in the middle of the neck,) I proceeded to repeat the experiments, by dividing the nerves near the base of the cranium, and above the origin of the superior laryngeals. The results, however, were the same as when the nerves were cut in the usual manner." p. 161.

Brachet's experiments appeared to prove, beyond doubt, that section of the pneumogastrics annihilated the *besoin de respirer.* Our author's experience is at direct variance with the French physiologist, and is doubtless correct. In numerous animals, when the access of air to the lungs was *suddenly* and *fully* prevented, great distress and anxiety resulted.

Spinal Accessory Nerve. Before discussing the functions of this nerve, Dr. Reid adverts to its anatomical arrangement, origin, and distribution. All that we shall be enabled to do, will be briefly to enumerate the conclusions, which, after numerous experiments, he is led to infer.

1. "The filaments of the *external* branch of the *spinal accessory* are principally motor, and that the sensitive filaments must be very few in number. Whether these filaments belong originally to the nerve, or whether it derives them from the other nerves, at the base of the cranium, with which it anastomoses, we cannot at present determine." p. 169.

2. "As far as we can observe, the functions of the external branch of the *spinal accessory*, exactly resemble those of the filaments coming from the cervical plexus, with which it anastomoses so frequently." p. 171.

3. "From these experiments, we think there can be no doubt that the trunk of the *par-vagum* contains within it *motor* filaments, independent of those which it receives from the *internal* branch of the spinal accessory; that the internal branch of the spinal accessory, assists in moving the muscles of the pharynx, we are satisfied, not only from the experiments just stated, but also, from those on the pharyngeal branch of the *par-vagum.* Of the probable destination and functions of the other filaments of the *internal* branch of the *accessory*, we cannot pretend to judge without more extended inquiries. We certainly do not consider that these experiments entitle us to assert that they are not motor filaments." p. 173.

The experiments of Dr. Reid, upon the glossopharyngeal and spinal accessory, oppose directly the functions assigned them by Sir Charles Bell, and shake to its very foundation his ingenious respiratory theory. If one associated movement can be maintained, without an especial tract of nervous matter, why may they not all be carried on simply through the interference of the voluntary nerves. No one pretends to have discovered an urinary, or a defecatory tract.

From the brief epitome of Dr. Reid's communication which we have been enabled to give, our readers will, we feel assured, justify the favorable opinion we expressed, at the onset, of our author's labours, and will be stimulated to a speedy and careful perusal of them. We hope to see, ere long, the whole essay, enriching the pages of some of our contemporaries, whose plan will allow of its entire translation.

---

Traité Pratique Des Maladies Vénériennes, *ou, Recherches Critiques et Experimentales sur l'Inoculation appliquée à l'étude de ces Maladies; Suivies d'un Résumé Thérapeutique et d'un Formulaire Spécial.* Par Ph. Ricord, Docteuren Médécine, Chirurgien de l'Hopital des Vénériens de Paris, &c. &c. Paris: 1838, pp. 808, 8vo.

A Practical Treatise on Venereal Diseases, *or, Critical and Experimental Researches upon Inoculation applied to the study of these Diseases,* &c. By Ph. Ricord, M. D., Surgeon to the Venereal Hospital at Paris, &c. &c. Paris: 1838, pp. 808, 8vo.

"The venereal disease arises from a poison, which, as it is produced by disease, is capable again of producing a similar disease," is the well known result of the experience and reflections of John Hunter. Among the attempts of modern times to simplify nosology, not the least conspicuous, and supported by respectable authority, have been the efforts directed against a belief in a specific contagious syphilitic virus. The anti-specific doctrines of the physiological school of medicine, have not, however, met with general favour. The faith of the mass of medical thinkers in the conclusion of Hunter, has been unshaken; they have always believed that syphilis must be traced to a special cause, having as distinct an existence, as the venom of hydrophobia, the poison of the viper, or the matter of small-pox. Much talent and ingenuity have, it is true, been enlisted in the advocacy of the contrary opinions, nor have they been abandoned. A disciple of Broussais, M. Desruelles,* has lately been in the field, in the defence of the non-specific doctrines. He has expended much research, and displayed some subtlety, in the endeavour to reconcile, with facts, the speculations of his master. "Physiological medicine," says Broussais, "should confine itself, in syphilis, to the study of the forms and degrees of *irritation*, in the different parts of the body, and to noting the modifications that may be opposed to them."

The conclusions of M. Ricord, based upon experiments, which he has been for a series of years pursuing in the Venereal Hospital of Paris, add new and irresistible weight to the belief in the existence of a distinct venereal virus. Following in the track of Hunter, he has applied *inoculation* to the study of venereal diseases, and his results and deductions are before us, in the volume just issued from the Parisian press. M. Ricord has, from time to time, put forth fugitive papers on these subjects, but his researches are now, for the first time, offered to the public in the collective form. Experiments similar to those of M. Ricord have been latterly made in Great Britain,* and with confirmatory results. We propose to offer to our readers a somewhat extended analysis of this remarkable and very interesting work.

The Traité Pratique offers three grand divisions: the first is devoted to a critical examination of the subject of inoculation, applied to the study of venereal affections; in the second, the author brings a mass of practical observations to the support of his opinions; the third is a therapeutic summary, or an account of the modes of treatment which he found most successful at the Venereal Hospital.

M. Ricord's experiments on inoculation were made upon individuals affected with the syphilitic disease. No circumstances can justify, he very properly admits, communicating to a healthy man a disease, the consequences of which it is impossible to foresee. All his objects were, besides, equally attainable by experimenting upon subjects, already exposed to the action of the virus. For, the existence of one primary venereal sore, is no obstacle to the contracting of others, which, moreover, cannot be developed, save by contagion, from contact with pus, from the first or a similar ulcer. Again: constitutional infection cannot prevent a fresh development of primary sores, nor is the occurrence of the former at all dependent upon the number of these at a given period. These positions are susceptible of easy demonstration, and, admitting them to be established, we pass with M. Ricord to his experimental investigations. "All the secretions, whether normal or morbid, of individuals reputed to be affected with syphilis, were examined by the mode of inoculation, and the results were invariable with those of but one form, which was the primitive ulcer, otherwise

*Traité Pratique des Maladies Vénériennes. Paris, 1836.

*By Mr. Thompson, (see London Medical and Surgical Journal, 1833,) and Mr. Wallace of Dublin, (see Lancet, 1837.)

termed chancre." p. 85. The distinction is made, that a chancre secretes the venereal virus, only while the ulceration is in a state of progress, or in *statu quo*, and that, with the first efforts towards cicatrization, the virus disappears. The location of a chancre has no influence on its special nature; it is independent of the genital organs.

The conclusion which M. Ricord deduces from his experiments, then, is, that "chancres, where ever situate, are the consequences of a specific pus which they alone secrete, and which, like a true leaven or special ferment, reproduces a disease identical with itself, whenever properly applied." p. 88.

The effects of the inoculation of the pus of a chancre beneath the epidermis, are the following.

During the first twenty-four hours, simple redness, as in vaccination, results; from the second to the third day, there is slight tumefaction, and the point of inoculation presents the aspect of a small papula, surrounded by a red areola; from the third to the fourth day, the epidermis is elevated by a liquid more or less turbid, and often takes a vesicular form, having, at its summit, a black spot, the result of the drying of the blood from the original puncture; from the fourth to the fifth day, the morbid secretion increases, becomes purulent, the pustular form makes its appearance, and a depression of its summit gives it an umbilicated aspect, resembling the pustule of variola. At this period, the areola, which has been extending itself, often begins to disappear; but, from the fifth day, the subjacent tissues, up to this time not affected, or merely slightly edematous, become infiltrated and hardened by the effusion of a plastic lymph, which gives to the touch the resisting and elastic feel of certain cartilages. Finally, from the sixth day, the pus thickens, the pustule shrivels, and incrustations begin to be formed. If they are not detached, they are seen to increase in size from their base, and, rising in strata, to take the form of a truncated cone with a depressed summit. If the incrustations be detached, or fall, an ulcer is found beneath, with the indurated base described, and a bottom, of a depth represented by the entire thickness of the skin, with a white surface of a grayish tint, composed of a lardaceous matter, sometimes pultaceous, or even with a false membrane, not to be detached by any cleansing process. The edges of the ulcer, at this period, are as smoothly cut as if by a circular instrument; they are, however, insulated, to a greater or less extent, and exhibit slight indentations, and a surface like that of the bottom; their margin is indurated like the base, and presents a sort of ring, of a reddish-brown colour, with a violet tint, which, projecting above the surrounding parts, elevates and slightly turns down the edges, giving the ulcers at first an infundibuliform appearance.

We have thus minutely described these phenomena of inoculation, because, upon their regular and constant occurrence the author bases the establishment of a general rule, from which he makes his most important deductions. The most striking of these deductions, is, that no external appearances can determine, with certainty, the existence of a chancre, the only absolute test being the pus which it secretes, which always remains identical. Our author has here, perhaps, the appearance of reasoning in a vicious circle; he has told us that the matter of chancres, when inoculated, produces certain invariable results; and yet, we are to know a chancre only by the occurrence of these results. This seeming discrepancy, however, disappears, if we give credit to the assertion, that regular, characteristic syphilitic constitutional affections are the sequelæ only of chancre, or hereditary transmission. For confirmation of these two important propositions, M. Ricord appeals to the results of the experiments *publicly* performed by him, during a course of years, at his hospital at Paris. We are disposed to believe his facts, and to admit with him, first, that, if out of a series of ulcers upon the genital organs and other parts, inoculation with the matter of a given number was attended with an unvarying succession of phenomena, the existence of a specific local affection is proved; and, secondly, that if constitutional symptoms occur, only after ulcers of this special character, the existence of a general, as well as local specific disease is likewise demonstrated.

Among M. Ricord's other conclusions, we find the following. The pus of a chancre can alone produce a chancre, and the best mode of producing it is inoculation with the lancet; it may be produced without venereal or other excitation of the parts acted on; inoculation never fails, if the pus be properly selected and applied; pus taken from an inoculated pustule reproduces a chancre, and so on in an infinite series; chancre, at its commencement, is a local affection; if it be completely destroyed by caustic during the three, four, or five days, immediately following the application of the cause, there is no risk of secondary inflammation; the induration of chancres commences about the fifth day, this may be considered to denote that the virus is penetrating into the economy, and that the chances of secondary symptoms are increased.

After these general considerations on the subject of inoculation, (upon which we have dwelt at some length,) the author proceeds to treat of it, with reference, successively, to primary and secondary symptoms, therapeutics, hygiene, and medical jurisprudence.

It may be supposed, that inoculation is invoked as the only means of distinguishing true syphilitic primary sores from other affections, reputed to be of that nature, because noticed after impure sexual intercourse. These may be, we are told, blennorrhagia, in its various seats, bubo, viewed as a primary affection, and the mucous tubercle, styled primitive. No notice is taken of ulcers on the genitals, from laceration, excoriation, or other such cause, which are often not a little intractable in their character, and may, we think, be confounded with chancre. Inoculation must here be a valuable means of diagnosis.

From the previous conclusions of our author, it will be inferred, that his experiments resulted in establishing, contrary to the views of Hunter, that blennorrhagia has no identity with chancre, and is incapable of generating it. Blennorrhagia is an affection of a catarrhal character, usually, indeed, to be traced to the irritating application of such a discharge to the part implicated, but distinct from the virulent pus of a chancre. But a woman with a blennorrhagia, it is objected, has been known to communicate to one man a running from the urethra, and to another a chancre. The application, however, of the speculum, by M. Ricord, to the study of venereal diseases, has solved this difficulty. Deep-seated chancres of the vagina and uterus, are found, by the aid of this instrument, to co-exist with the outward appearances of simple blennorrhagia. In all the cases, and they were numerous, in which women, who had communicated syphilis, were thus examined by M. Ricord, ulcers of the genitals were detected. It may be fairly inferred, that, in the instance above cited, there was the same combination that presented itself at the Venereal Hospital. If, to the above facts, we add, that constitutional syphilis was never found by M. Ricord, to be the consequence of blennorrhagia, his conclusion must be allowed, that chancre and blennorrhagia constitute two entirely distinct diseases.

On the subject of buboes, M. Ricord makes the generally admitted distinction of buboes from absorption, and from sympathy. The bubo arising from absorption of the pus of a chancre, he considers virulent and capable of producing a chancre, by inoculation. This, too, he looks upon, as the only final test of the character of a bubo. The vexed question, can there be primary syphilitic buboes, M. Ricord answers in the affirmative, though such accidents he deems of exceeding infrequency of occurrence. For them to take place, the lymphatics must necessarily have their mouths opening upon the mucous, or cutaneous surface; absorption, in this case, cannot be preceded by any sort of imbibition, as the tissues, impregnated with pus of a chancre, must become infected, and as a necessary consequence, take on the ulcerated process.

The nature of certain secondary syphilitic sores, occasionally mistaken for primary, as the mucous tubercle, (venereal wart,) is briefly discussed by M. Ricord. He determines them not to be primary, to be incapable of communication by inoculation, and, like other constitutional symptoms, to be transmitted only by the mode of inheritance.

Our author's classification of the symptoms of syphilis are, into primary, successive, secondary, and tertiary, to which he adds non-syphilitic affections, the development of which has been favored by the occurrence of syphilis. The primitive accident is, of course, the chancre; the successive are such, as arise from the simple extension of the first local symptom, as, new chancres, inflammatory or virulent abscesses, and the like; secondary symptoms are those produced by constitutional infection, showing themselves on the skin, mucous membrane, eyes, testicles, &c.; they are not susceptible of inoculation, but are, incontestably, transmitted as an inheritance, from parent to child; tertiary accidents occur long after the disappearance of the primitive, and, most generally, after the occurrence of secondary symptoms; they are incapable of hereditary transmission, but are a frequent cause, through generation, of scrofulas, which the author looks upon as often degenerate syphilis. These tertiary symptoms are nodes, deep-seated tubercles of the brain, cellular tissue, &c., diseases of the bones and periosteum, and other internal affections not yet well defined.

The venereal virus is carried into the system by venous absorption. As this is distinct from the lymphatic absorption which produces buboes, there is no necessary connection between the appearance of these and constitutional infection. Once united to the blood, however, the virus loses the power of inoculation, inoculable pus having never been detected in the veins. Secondary symptoms cannot, therefore, be inoculated; but ulcers on other parts than the genitals, as the lips, tongue, and even pharynx, on all of which M. Ricord has seen primitive sores, arising from unnatural intercourse, should not be confounded with constitutional symptoms.

The results of the application of inoculation to the study of therapeutics are, the author admits, still meagre. He is sanguine, that an absolute prophylaxis against syphilis will one day be discovered, and dwells upon the value of inoculation as a test for the employment of any future specific remedy for syphilis. The usefulness of inoculation, as a means of prognosis, need not be urged. The importance, too, of its application to hygiene and medical jurisprudence, cannot be too highly estimated.

The second part of the Traité Pratique contains, as we have mentioned, a summary of the author's clinical and experimental observations. They were made between the years 1831–'37. During this period, a mass of results was obtained, sufficient, we think, to authorise the important deductions which are arrived at. Connected with this subject, the author's credibility is the most important question to be discussed. The assertions of French writers are, we know, received in this country with some distrust. The flat contradiction which the published statements of a distinguished Parisian Surgeon, M. Lisfranc, received from the internes of his hospital, is fresh in the recollection of our readers. But we are disposed to yield credence to M. Ricord, because we think his facts bear with them the evidence of their veracity. They are consistent with themselves; and the absence of discrepancy in statements adds strong weight to testimony. The results of his experimental investigations will moreover b[illegible]nfirmed by the individual observations, and a [illegible]*ri* reasoning of the profession generally. [illegible]Ricord has, we believe, a fair character for professional honour. He is by birth an American, a native of Baltimore, where his boyhood was spent.

(*To be concluded in our next.*)

